# Hypophosphatemia after high-dose iron repletion with ferric carboxymaltose and ferric derisomaltose—the randomized controlled HOMe aFers study

**DOI:** 10.1186/s12916-020-01643-5

**Published:** 2020-07-13

**Authors:** I. E. Emrich, F. Lizzi, J. D. Siegel, S. Seiler-Mussler, C. Ukena, D. Kaddu-Mulindwa, R. D’Amelio, S. Wagenpfeil, V. M. Brandenburg, M. Böhm, D. Fliser, G. H. Heine

**Affiliations:** 1 Internal Medicine III—Cardiology, Angiology and Intensive Care Medicine, Saarland University Medical Center, Homburg, Germany; 2 Internal Medicine IV—Nephrology and Hypertension, Saarland University Medical Center, Homburg, Germany; 3 VAUBAN Praxis Saarlouis, Saarlouis, Germany; 4 Internal Medicine I—Hematology and Oncology, Saarland University Medical Center, Homburg, Germany; 5 Institute for Medical Biometry, Epidemiology and Medical Informatics, University Medical Center, Saarland University, Campus Homburg, Homburg, Germany; 6 Department of Cardiology and Nephrology, Rhein-Maas Klinikum, Würselen, Germany; 7 AGAPLESION MARKUS KRANKENHAUS, Frankfurt am Main, Germany

**Keywords:** Hypophosphatemia, FGF23, Iron deficiency anemia, Ferric carboxymaltose, Ferric derisomaltose

## Abstract

**Background:**

In patients with iron deficiency anemia, ferric carboxymaltose (FCM) and ferric derisomaltose (FDI) allow high-dose iron repletion. While FCM is reported to induce hypophosphatemia, the frequency of hypophosphatemia after an equivalent dosage of FDI had not been assessed prospectively.

**Methods:**

In the prospective, single-center, double-blind *HOMe aFers* study, 26 women with iron deficiency anemia (hemoglobin < 12 g/dL plus either plasma ferritin ≤ 100 ng/mL or a plasma ferritin ≤ 300 ng/mL and transferrin saturation (TSAT) ≤ 30%) were randomized to a single intravenous infusion of 20 mg/kg body weight (up to a maximum of 1000 mg) FCM or FDI. The primary endpoint was the incidence of hypophosphatemia (plasma phosphorus levels < 2.0 mg/dL at day 1, day 7 ± 2, and/or day 35 ± 2 after the infusion). In order to investigate potential skeletal and cardiovascular implications, we assessed changes in other components of mineral and bone metabolism, left ventricular function, and arrhythmias.

**Results:**

Hypophosphatemia occurred more frequently in women treated with FCM (9 out of 12 [75%]) than in those treated with FDI (1 out of 13 [8%]; *p* = 0.001). Within 24 h after iron supplementation, women in the FCM group had significant higher plasma intact FGF23 (*p* < 0.001) and lower plasma 1.25-dihydroxyvitamin D (*p* < 0.001). As an indicator of urinary phosphorus losses, urinary fractional phosphorus excretion was higher in the FCM group (*p* = 0.021 at day 7 ± 2 after iron supplementation). We did not observe differences in skeletal and cardiovascular markers, potentially because of the limited number of participants.

**Conclusions:**

While both FCM and FDI provide efficient iron repletion in participants with iron deficiency anemia, FCM induced hypophosphatemia more often than FDI.

**Trial registration:**

Clinical Trials.gov NCT02905539. Registered on 8 September 2016.

2015-004808-36 (EudraCT Number)

U1111-1176-4563 (WHO Universal Trial Number)

DRKS00010766 (Deutsches Register Klinischer Studien)

## Background

Approximately 2.2 billion people suffer from anemia worldwide. Iron deficiency is the most frequent cause of anemia [1], and menstruating women [2] are particularly affected. The importance of adequate iron repletion is undisputed, and in the majority of individuals without chronic disease, treatment with oral iron compounds is sufficient [3]. When intolerance or inefficacy precludes success of oral iron, intravenous administration is indicated [4].

Several novel iron compounds have been approved that allow the repletion of large amounts of iron with a single intravenous infusion [5]. Ferric carboxymaltose (FCM) and ferric derisomaltose (FDI) are widely used, but FCM treatment has been reported to be associated with hypophosphatemia 2 to 5 weeks after administration, usually resolving spontaneously after 6 to 12 weeks [6]. Hypophosphatemia after FCM treatment is caused by transiently rising plasma levels of the phosphaturic hormone intact FGF23 (iFGF23), which stimulates the renal excretion of phosphorus. Such transient increase of iFGF23 with a consecutive period of hypophosphatemia after FCM has been described to induce relevant clinical consequences in retrospective case reports [7].

Interestingly, in randomized clinical trials, patients who received iron dextran [8] or ferumoxytol [6] rather than FCM at equivalent dosages did not experience hypophosphatemia, as iron dextran and ferumoxytol did not induce an increase in iFGF23 in these trials [6, 8].

Similarly, FCM caused hypophosphatemia substantially more often than FDI when both compounds are given at dosages licensed in the USA (FCM, 750 mg at day 0 and day 7; FDI, 1000 mg) [9].

In the *HOMe aFers* (HOMburg evaluations on application of Ferrum study 1) trial, we compared the incidence of hypophosphatemia in women randomized to receive either FDI or FCM at equivalent dosages, as licensed in Europe. Furthermore, we investigated changes in markers of mineral and bone metabolism, changes in left ventricular ejection fraction, and the incidence of arrhythmic events.

## Methods

In a prospective, randomized, single-center, double-blinded clinical trial, we compared the effects of a single intravenous infusion of either ferric carboxymaltose or ferric derisomaltose (also known as iron isomaltoside 1000) on phosphorus homeostasis.

### Study population

Adult women (18 years or older) with iron deficiency anemia due to uterine bleeding in whom oral iron repletion was not tolerated or not efficient were invited to participate in our *HOMe aFers* trial.

Anemia was defined following the WHO criteria as hemoglobin (Hb) < 12 g/dL. In line with earlier studies [8], we defined iron deficiency anemia as anemia plus either a serum ferritin ≤ 100 ng/mL or a serum ferritin ≤ 300 ng/mL and transferrin saturation (TSAT) ≤ 30%.

Exclusion criteria included advanced chronic kidney disease (estimated glomerular filtration rate [eGFR] according to the creatinine-based CKD-EPI equation ≤ 15 mL/min/1.73 m^2^ or renal replacement therapy), hypophosphatemia (plasma phosphorus < 2.5 mg/dL at screening), pregnancy, lactation, or a known hypersensitivity to any intravenous iron preparations. Further exclusion criteria are listed in the supplement (Additional file 1: Table S1). The study was approved by the local ethic committee (119/16) and registered at Clinical Trials.gov (NCT02905539), in agreement with the declaration of Helsinki (2013). All participating women gave written informed consent.

The study’s design and flow are presented in figure S1 A (Additional file 2: Figure S1A) and in the supplement (Additional file 3: Table S2). After a screening visit (visit 1), eligible participants were randomized by a computer-based system to receive either FCM or FDI (20 mg/kg up to a maximum of 1000 mg), which they received at visit 2. Both iron compounds were diluted in 250 mL saline solution and infused over 30 min.

Follow-up visits were scheduled directly 1 day after iron supplementation (visit 3), at day 7 ± 2 (visit 4), and at day 35 ± 2 (visit 5) in order to detect clinical and laboratory changes.

All patients were instructed to contact the study center immediately in case of any potential side effects.

### Endpoints

The primary study endpoint was defined as the incidence of hypophosphatemia (plasma phosphorus levels < 2.0 mg/dL) during any of the three follow-up visits.

As secondary endpoints, we analyzed changes in plasma phosphorus, urinary fractional excretion of phosphorus (FePi), serum 25-hydroxyvitamin D3 and serum 1.25-dihydroxyvitamin D, plasma intact and c-terminal FGF23 (cFGF23), plasma calcium and parathyroid hormone (PTH), plasma alkaline phosphatase, plasma hepcidin-25, serum n-terminal propeptide of type I collagen (PINP), urinary pyridinoline (PYD), urinary desoxypyridinoline (DPD), QT interval and QT dispersion in 12-lead electrocardiogram (ECG), and left ventricular mass index (LVMI), left atrial volume index (LAVI), left ventricular ejection fraction (LVEF), and diastolic left ventricular function (E/e’) by echocardiography. Finally, we assessed the incidence of (supra) ventricular cardiac arrhythmia in Holter ECG. We additionally assessed the quality of life by Short Form 36 Health Survey (SF-36) [10], functional impairment by Sheehan disability scale [11], and fatigue by the German version of The Multidimensional Fatigue Inventory (MFI) [12] with standardized questionnaires, the results of which will be presented separately.

### Laboratory tests

Blood samples were drawn under standardized conditions, and routine laboratory parameters were analyzed at the Department of Laboratory Medicine of the Saarland University Medical Center. Serum 1.25-dihydroxyvitamin D was measured by an in vitro chemiluminescence immunoassay (DiaSorin, Stillwater, MN, USA), PINP by ELICA (Roche Diagnostics, Mannheim, BW, Germany), and PYD and DPD by HPLC (UltiMate 3000, ThermoFisher Scientific, Waltham, MA, USA). iFGF23, cFGF23, and Hepcidin-25 were measured by ELISA (iFGF23 and cFGF23: second-generation ELISA; Immuntopics International, San Clemente, CA, USA, and Hepcidin-25: DRG international.inc, Springfield Township, NJ, USA), and intact PHT was measured by a second-generation electro-chemiluminescence immunoassay (Hoffmann-LA Roche, Basel, Switzerland).

Urinary fractional excretion of phosphorus (FePi) was calculated from a spot urine sample as

$$ \frac{\mathrm{urine}\ \mathrm{phosphorus}\ \left[\frac{\mathrm{mg}}{\mathrm{dL}}\right]\times \mathrm{plasma}\ \mathrm{creatinine}\left[\frac{\mathrm{mg}}{\mathrm{dL}}\right]}{\mathrm{plasma}\ \mathrm{phosphorus}\ \left[\frac{\mathrm{mg}}{\mathrm{dL}}\right]\times \mathrm{urine}\ \mathrm{creatinine}\left[\frac{\mathrm{mg}}{\mathrm{dL}}\right]}\times 100 $$

### Further clinical characteristics

Twelve-lead ECGs were documented after 5 min of rest in a lying position at visits 1, 2, 4, and 5 and analyzed by an independent physician using Dr. Gerhard Schmidt PC-EKG SmartScript, version 1.10R060, Neunkirchen, Germany. Holter ECG recordings were performed at visits 1 and 4 and analyzed by an independent investigator using the software Pathfinder© (Spacelabs Healthcare, Washington D.C., USA). From Holter ECG recording, supraventricular and ventricular arrhythmias were counted. Echocardiography was performed and analyzed according to the guidelines of the American Society of Echocardiography (ASE) [13] using a Sequoia C512 Ultrasound Unit (Acuson, Thousand Oaks, CA, USA) with a linear probe (model 3V2c; 2–3 MHz). From standard parasternal and apical views, LVMI and LAVI were calculated according to the ASE guidelines [13]. As a parameter of diastolic left ventricular function (E/e’), we assessed the ratio of early diastolic mitral inflow velocity (E; assessed with pulsed wave Doppler ultrasound) to early diastolic septal mitral annular velocity (e’; assessed with tissue Doppler recording). Systolic left ventricular function was assessed as left ventricular ejection fraction (LVEF) and by visual inspection.

### Statistical analysis

Data management and statistical analysis were performed with SPSS Statistics 25 (IBM, SPSS Statistics 25).

Continuous data are expressed as mean ± SD, and the two groups were compared using *t* test for independent samples. In case of skewed distribution, levels were presented as median (interquartile range) and comparison is due to the Mann-Whitney *U* test. Categorical data are expressed as absolute numbers as well as relative frequencies, and the two groups were compared using Fisher’s exact test. The correlation between parameters of iron metabolism and parameters of bone and mineral metabolism was assessed by Pearson correlations. Comparison over time was performed by ANOVA with repeated measures or the Friedman test, as appropriate. Relative changes from baseline to each individual follow-up time point between the two treatment arms were calculated and compared by *t* test for two independent samples. Differences in the incidence of hypophosphatemia between the two treatment arms were assessed by Fisher’s exact test.

**Fig. 1:**
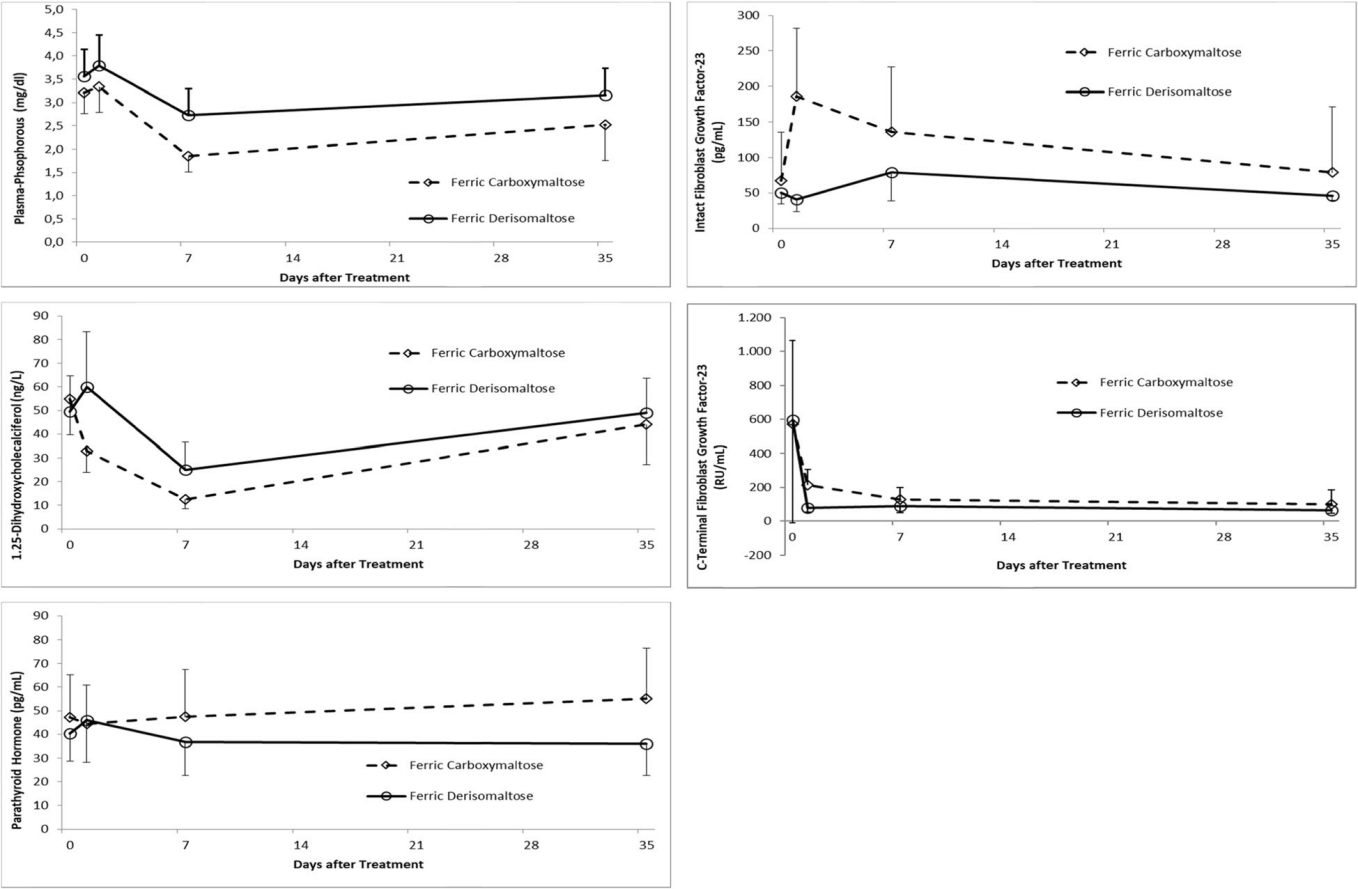
Changes of parameters of bone and mineral metabolism in both treatment arms over time after intravenous iron repletion up to day 35 ± 2. Mean and standard deviation are given for every single time point (see whiskers in graphics)

We initially planned to randomize 60 patients, but a priori decided to include an interim analysis after inclusion of 30 patients, with the option to stop the study if the primary endpoint was prematurely met. For logistic reasons, we decided to perform this interim analysis after 26 rather than 30 patients, as patient recruitment was slower than anticipated.

Group sample sizes of 30 in group 1 and 30 in group 2 achieve at least a power of 80% to detect a difference between the group proportions of 40% with the two-sided Fisher’s exact test and a significance level of 0.05. Based upon an earlier interventional trial by Wolf et al. [8], we assumed the proportion of hypophosphatemia with FCM to be ~ 0.60. Thus, under the null hypothesis (no difference between FCM and FDI), the proportion of hypophosphatemia with FDI were 0.60, and it was 0.20 under the alternative hypothesis.

Power analyses were performed with PASS 2019 Power Analysis and Sample Size Software (2019); NCSS, LLC, Kaysville, UT, USA, ncss.com/software/pass.

Two-sided *p* values < 0.05 were considered significant.

## Results

From August 2016 to August 2018, a total of 32 women were screened, six of whom did not meet inclusion criteria. Consecutively, 26 women with iron deficiency anemia due to uterine bleeding were included and randomized to FCM (*n* = 13) or to FDI (*n* = 13). One woman who was randomized to FCM withdrew her informed consent after receiving the study drug, but before the final study visit. All participants received a single infusion of 1000 mg either FCM or FDI, as all patients had 50 kg of body weight or more.

### Baseline characteristics

Mean age was 40 ± 10 years in the FCM group and 34 ± 11 years in the FDI group. All participants were Caucasians; six participants in the FCM group and five participants in the FDI group had received previous intravenous iron before study initiation. At baseline, systolic blood pressure was significantly higher (*p* = 0.022) and serum 25-hydroxyvitamin D3 levels were significantly lower (*p* = 0.026) in the FCM treatment group than in the FDI group; other baseline characteristics did not differ significantly between the two treatment groups (Table 1).

**Table 1:** Baseline characteristics

	FCM (*n* = 13)	FDI (*n* = 13)	*p* value
**Demographics**			
Age [years]	40 ± 10	34 ± 11	0.166
Caucasians, *n* [%]	13 (100)	13 (100)	
eGFRCrea (CKD-EPI) [mL/min/1.73 m^2^]	102 ± 11	101 ± 21	0.951
BMI [kg/m^2^]	26 ± 6	23 ± 2	0.140
Diabetes mellitus, *n* [%]	1 (7.7)	0 (0)	0.308
**Vital signs**			
BP systolic [mmHg]	124 ± 10	114 ± 10	**0.022**
BP diastolic [mmHg]	79 ± 8	74 ± 10	0.217
Heart rate [BPM]	71 ± 12	69 ± 12	0.667
**Iron deficiency parameters**			
Previous intravenous iron therapy, *n* [%]	6 (46.2)	5 (38.5)	0.691
Hemoglobin [g/dL]	10.1 ± 1.4	10.7 ± 1.2	0.273
Transferrin [mg/dL]	344 ± 37	325 ± 34	0.186
Transferrin saturation [%]	4 [3; 7]	5 [4; 7]	0.644
Ferritin [ng/mL]	6 [4; 8]	8 [6; 12]	0.193
Hepcidin [ng/mL]	0.9 [0.4; 1.4]	0.6 [0.1; 1.1]	0.469
**Mineral metabolism parameters**			
Plasma phosphorus [mg/dL]	3.2 ± 0.4	3.6 ± 0.6	0.101
FePi [%]	12 ± 6	14 ± 9	0.417
Plasma intact FGF23 [pg/mL]	49 [42; 60]	47 [40; 58]	0.387
Plasma c-terminal FGF23 [RU/mL]	385 [199; 998]	451 [190; 840]	0.911
Serum calcium [mmol/L]	2.3 ± 0.1	2.3 ± 0.1	1.000
Serum parathormone [pg/mL]	40 [37; 55]	40 [33; 52]	0.253
Serum 1.25-dihydroxyvitamin D [ng/L]	55 ± 15	51 ± 15	0.378
Serum 25-hydroxyvitamin D3 [ng/mL]	19 [10; 22]	24 [21; 29]	**0.026**
Alkaline phosphatase [U/L]	74 [52; 82]	48 [41; 91]	0.724
Serum prokollagen type 1 N propeptide [μg/L]	51 [32; 63]	57 [40; 74]	0.305
Urinary pyridinoline [μg/g creatinine]	176 [138; 187]	176 [153; 236]	0.183
Urinary desoxypyridinoline [μg/g creatinine]	36 [31; 42]	46 [27; 58]	0.303
**Clinical safety parameters**			
LVMI [g/m^2^]	69 ± 13	67 ± 10	0.693
LAVI [mL/m^2^]	42 ± 10	38 ± 6	0.308
E/e’	6.9 ± 1.5	6.0 ± 1.4	0.146
LVEF [%]	62 ± 8	61 ± 5	0.833
Heart rate [BPM]	77 ± 9	79 ± 11	0.631
SVES [*n*]/24 h	2 [0; 6]	4 [0; 52]	0.349
VES [*n*]/24 h	8 [0; 37]	2 [0; 8]	0.197
corrected QT interval [ms]	406 ± 20	403 ± 22	0.768
QT dispersion [ms]	45 ± 18	44 ± 17	0.899

Continuous variables are presented as mean ± standard deviation or median ± interquartile range as appropriate; categorial variables are presented as absolute numbers and percentage. *p* values in bold letters are significant. *FCM* ferric carboxymaltose, *FDI* ferric derisomaltose, *eGFRCrea (CKD-EPI)* estimated glomerular filtration rate (according to the CKD-EPI equation; “Chronic Kidney Disease Epidemiology Collaboration”), *BMI* body mass index, *BP systolic* systolic blood pressure, *BP diastolic* diastolic blood pressure, *FGF23* fibroblast growth factor 23, *FePi* urinary fractional phosphorus excretion, *LVMI* left ventricular mass index, *LAVI* left atrial volume index, *E/e’* diastolic left ventricular function, *LVEF* left ventricular ejection fraction, *SVES* supraventricular extrasystole, *VES* ventricular extrasystole

No participating woman had hypophosphatemia at baseline; moreover, all women had fractional excretion of phosphorus, plasma calcium, and plasma PTH within the physiological range.

In iron-deficient women, baseline plasma cFGF23 levels were substantially elevated [FCM 385 [199; 998] RU/mL vs. FDI 451 [190; 840] RU/mL; *p* = 0.911], and plasma iFGF23 levels were within the normal range [FCM 49 [42; 60] pg/mL vs. FDI 47 [40; 58] pg/mL; *p* = 0.387].

Baseline plasma cFGF23 correlated moderately inversely with TSAT (*r* = − 0.403; *p* = 0.046), and weakly inversely with ferritin (*r* = − 0.305; *p* = 0.138), while iFGF23 correlated neither with TSAT nor with ferritin (Additional file 4: Table S3).

### Endpoints

The predefined primary endpoint of hypophosphatemia (plasma phosphorus < 2 mg/dL) at any post-infusion study visit occurred in 75% (9 of 12) of the women in the FCM group and 8% (1 of 13) in the FDI group (*p* = 0.001) (Additional file 5: Table S4).

After a mild increase of plasma phosphorus within the first 24 h after iron supplementation in both groups, a decrease in plasma phosphorus compared to baseline was observed in both groups (ANOVA with repeated measures: *p* < 0.001), with numerically lowest values at visit 4 (Fig. 1). This decrease in plasma phosphorus was accompanied by an increase in FePi, reaching its numeric maximum again at visit 4 in both groups. The changes in FePi were significantly more prominent within the FCM group than within the FDI group (comparison of changes from baseline to V4 between the two treatment groups: *p* = 0.021; Table 2).

**Table 2:** Parameters of iron, bone, and mineral metabolism

	A										B		
	FCM					FDI					*p* values of group comparison for relative changes to V2		
	V2	V3	V4	V5	*p*	V2	V3	V4	V5	*p*	*p* [V3]	*p* [V4]	*p* [V5]
Phosphorus [mg/dL]	3.2 ± 0.4	3.3 ± 0.5	1.9 ± 0.3	2.5 ± 0.8	**< 0.001**	3.6 ± 0.6	3.8 ± 0.7	2.7 ± 0.6	3.2 ± 0.6	**< 0.001**	0.537	**0.005**	0.249
FePi [%]	12 ± 6	14 ± 7	22 ± 10	15 ± 10	**0.005**	14 ± 9	12 ± 8	16 ± 8	11 ± 7	**0.040**	0.274	**0.021**	0.253
iFGF23 [pg/mL]	49 [42; 60]	169 [131; 201]	103 [87; 166]	57 [37; 70]	**< 0.001**	47 [40; 58]	40 [33; 53]	79 [47; 103]	47 [43; 51]	0.140	**< 0.001**	0.187	0.191
cFGF23 [RU/mL]	385 [199; 998]	192 [138; 317]	103 [79; 183]	65 [44; 122]	**0.012**	451 [190; 840]	79 [71; 88]	96 [55; 107]	61 [54; 76]	**0.012**	**0.049**	0.177	0.733
Calcium [mmol/L]	2.3 ± 0.1	2.3 ± 0.1	2.3 ± 0.1	2.3 ± 0.1	0.643	2.3 ± 0.1	2.3 ± 0.1	2.3 ± 0.1	2.3 ± 0.1	0.602	0.900	0.237	0.876
PTH [pg/mL]	40 [37; 55]	43 [31; 54]	42 [36; 53]	47 [41; 68]	0.231	40 [33; 52]	44 [34; 56]	35 [23; 48]	34 [26; 47]	0.114	0.194	0.128	**0.018**
1.25-dihydroxyvitamin D [ng/L]	55 ± 15	33 ± 9	13 ± 4	44 ± 17	**< 0.001**	51 ± 15	62 ± 24	25 ± 12	51 ± 14	**< 0.001**	**< 0.001**	**0.002**	0.159
25-Hydroxyvitamin D3 [ng/mL]	19 [10; 22]	18 [9; 25]	16 [11; 27]	12 [9; 26]	0.498	24 [21; 29]	25 [19; 32]	25 [21; 36]	23 [16; 29]	**0.042**	0.902	0.447	0.200
Alkaline phosphatase [U/L]	74 [52; 82]	66 [46; 79]	75 [48; 90]	69 [46; 86]	0.334	48 [41; 91]	51 [39; 85]	57 [40; 73]	48 [40; 75]	0.196	0.565	0.557	0.351
Hepcidin-25 [ng/mL]	0.9 [0.4; 1.4]	2.5 [1.6; 6.7]	26.5 [3.3; 49.6]	8.6 [3.9; 25.9]	**0.045**	0.6 [0.1; 1.1]	8.0 [1.6; 24.1]	17.8 [2.7; 54.6]	6.7 [2.2; 36.6]	**0.025**	0.342	0.929	0.924
PINP [μg/L]	51 [32; 63]	n.a.	35 [28; 47]	40 [26; 54]	**0.001**	57 [40; 74]	n.a.	41 [30; 49]	50 [33; 83]	**0.001**	n.a.	0.903	0.866
PYD [μg/g creatinine]	176 [138; 187]	n.a.	168 [126; 191]	163 [142; 193]	0.651	176 [153; 236]	n.a.	147 [133; 163]	207 [148; 258]	0.255	n.a.	0.372	0.693
DPD [μg/g creatinine]	36 [31; 42]	n.a.	36 [24; 45]	39 [25; 46]	0.563	46 [27; 58]	n.a.	31 [25; 50]	46 [30; 60]	0.386	n.a.	0.939	0.517

Indicated are parameters of iron and bone metabolism at the four study visits of participants subdivided into the two treatment arms as well as relative changes between the two treatment arms between follow-up visits and the baseline visit. Presented are mean ± standard deviation or median and interquartile range, as appropriate. Longitudinal changes during the study period were first calculated within each group separately using ANOVA with repeated measures or the Friedman test, as appropriate (part A). Secondly, relative changes from baseline to each individual follow-up time point were calculated and compared between the two treatment arms by *t* test for two independent samples (part B). *FCM* ferric carboxymaltose, *FDI* ferric derisomaltose, *FePi* fractional urinary phosphorus excretion, *cFGF23* c-terminal FGF23, *iFGF23* intact FGF23, *PTH* parathyroid hormone, *PINP* serum n-terminal propeptide of type I collagen, *PYD* urinary pyridinoline, *DPD* urinary desoxypyridinoline. Significant values are given in bold. *V2* visit 2 (day 0 = baseline), *V3* visit 3 (day 1), *V4* visit 4 (day 7 ± 2), *V5* visit 5 (day 35 ± 2)

A significant rise of plasma iFGF23 was observed in the FCM group (*p* < 0.001), but not in the FDI group. In contrast, mean plasma cFGF23 dropped after the iron infusion in both study groups (*p* = 0.012 for both groups) (Fig. 1). No significant changes were observed for plasma PTH and for plasma calcium; a minor, non-significant increase of PTH with FCM and a minor fall with FDI rendered the between-group comparison at visit 5 significant. In both study groups, plasma 1.25-dihydroxyvitamin D decreased significantly (*p* < 0.001 for both groups); this decrease was more pronounced within the FCM group (comparison of changes from baseline to V3 and from baseline to V4 between the two treatment groups: *p* < 0.001 and *p* = 0.002, respectively; Table 2). 25-Hydroxyvitamin D levels did not change significantly over time in the FCM group, but in the FDI group (*p* = 0.042); however, the relative changes from baseline to any follow-up time point did not differ between the treatment arms. Hepcidin-25 increased significantly in both treatment arms after iron repletion (*p* = 0.045 in the FCM group; *p* = 0.025 in the FDI group; Table 2), while plasma PINP decreased in both treatment arms (*p* = 0.001; Table 2). Alkaline phosphatase, PYD, and urinary desoxypyridinoline did not change over time (Table 2).

Finally, no difference in the relative changes of echocardiographic and ECG parameters from baseline to V4 was found between the two treatment arms (Table 3).

**Table 3:** Parameters of echocardiography, Holter ECG, and 12-lead ECG

	A				B
	FCM		FDI		*p* values of group comparison for relative changes to V2
	V2	V4	V2	V4	*p*
**Echocardiography**					
LVMI [g/m^2^]	69 ± 13	63 ± 12	67 ± 10	60 ± 9	0.843
LAVI [mL/m^2^]	42 ± 10	46 ± 9	38 ± 6	39 ± 6	0.960
E/e’	6.9 ± 1.5	6.1 ± 1.3	6.0 ± 1.4	6.0 ± 1.3	0.089
LVEF [%]	62 ± 8	64 ± 5	61 ± 5	64 ± 4	0.892
**Holter ECG**					
Heart rate [BPM]	77 ± 9	73 ± 11	79 ± 11	80 ± 11	0.256
SVES [*n*]/24 h	2 [0; 6]	5 [0; 22]	4 [0; 52]	6 [2; 14]	0.117
VES [*n*]/24 h	8 [0; 37]	7 [1; 15]	2 [0; 8]	3 [0; 7]	0.177
**12-lead ECG**					
Corrected QT interval [ms]	406 ± 20	425 ± 19	403 ± 22	414 ± 22	0.064
QT dispersion [ms]	45 ± 18	42 ± 19	44 ± 17	40 ± 9	0.862

Indicated are parameters of echocardiography, Holter ECG, and 12-lead ECG at two study visits of participants subdivided into the two treatment arms as well as relative changes between the two treatment arms between follow-up visits and the baseline visit. Presented are mean ± standard deviation (part A) or median and interquartile range, as appropriate. Relative changes from baseline to each individual follow-up time point were calculated and compared between the two treatment arms by *t* test for two independent samples (part B). *FCM* ferric carboxymaltose, *FDI* ferric derisomaltose, *LVMI* Left ventricular mass index, *LAVI* Left atrial volume index, *E/e’* diastolic left ventricular function, *LVEF* left ventricular ejection fraction, *SVES* Supraventricular extrasystoles, *VES* ventricular extrasystoles, *n* absolute numbers, *BPM* beats per minute, *V2* visit 2 (day 0 = baseline), *V4* visit 4 (day 7 ± 2)

## Discussion

In *HOMe aFers*, FCM dosed intravenously as a single 1000mg infusion induced a larger reduction in plasma phosphorus than a single equivalent dosage of FDI. With respect to the primary study endpoint, 75% of women treated with FCM developed hypophosphatemia with plasma phosphorus levels below 2.0 mg/dL, compared to 8% in the FDI group. Hypophosphatemia was first observed at day 8 and plasma phosphorus levels normalized not later than days 35 to 37 in the majority of affected participants. After a mild increase of plasma phosphorus within the first 24 h after iron supplementation in both groups (which was previously also seen by Wolf et al. [8]), of which the etiology remains speculative, the drop in plasma phosphorus was evidently caused by renal phosphorus loss due to increased activity of the phosphaturic hormone iFGF23, as reflected by inappropriately high urinary fractional phosphorus excretion.

The magnitude and the time course of endocrinologic alterations in women with uterine bleeding receiving FCM were in line with those reported in the randomized clinical trials conducted by Wolf et al. [6, 8, 9]. In these three earlier trials as well as in the present study, iron-deficient patients had elevated plasma cFGF23 prior to repletion, and all iron compounds caused a tremendous drop of cFGF23 by around 80% within the first days of treatment. cFGF23 measurements comprise a substantial proportion of physiologically inactive FGF23, so high plasma cFGF23 before iron repletion did not cause hypophosphatemia, nor did its drop after iron treatment affect plasma phosphorus.

Unlike cFGF23, iFGF23 was not increased in study participants before iron supplementation. In all four studies, FCM induced a selective rise in iFGF23, which was not seen after iron dextran [8], ferumoxytol [6], and FDI ([9] and present trial), respectively. As iFGF23 measurements selectively reflect biologically active hormone levels, its rising plasma levels after FCM are followed by increased urinary phosphorus excretion and a subsequent drop of serum phosphorus. As iFGF23 also inhibits vitamin D activation, FCM treatment is also followed by a drop of plasma 1.25 dihydroxyvitamin D. This may subsequently reduce gastrointestinal calcium absorption and lower plasma calcium levels. Both low plasma 1.25 dihydroxyvitamin D and low calcium may finally explain why plasma PTH tended to increase after FCM, which is in line with secondary hyperparathyroidism reported after FCM in other trials [6, 8].

The proposed effects of iron deficiency and its supplementation are backed by animal data: in rodent experiments, iron deficiency stimulates FGF23 transcription in osteocytes/osteoblasts and in parallel its intracellular degradation to inactive fragments. These fragments are captured by cFGF23, but not by iFGF23 measurements, so that the affected mice have high plasma cFGF23, normal plasma iFGF23, and physiologic phosphorus levels [14, 15]. While iron supplementation with most intravenous agents will reduce both over-production and over-degradation within osteocytes/osteoblasts (and accordingly lower cFGF23), it remains presently largely enigmatic why FCM selectively raises iFGF23 and causes hypophosphatemia. Wolf et al. have discussed that the specific carbohydrate moieties of different intravenous iron compounds might affect FGF23 degradation in the osteocyte or within the circulation in a compound-specific way [8]. Alternatively, FCM might induce ectopic production of FGF23 by the liver or the lymphatic system without a parallel increase in its degradation, leading to an isolated increase of plasma iFGF23 [8]. However, such hypotheses still await their proof.

The clinical implications of FCM-induced hypophosphatemia need to be further studied. While very low plasma phosphorus in severely ill patients may lead to neurological disorders, hemolytic anemia, immunological disorders, and—in intubated patients—prolonged weaning due to muscle weakness [16], the implications of less severe hypophosphatemia in otherwise healthy individuals are currently not fully known. While cohort studies and case reports (particularly in patients with inflammatory bowel disease) claim repetitive intravenous iron supplementation with FCM with long-term hypophosphatemia to affect bone health [17], such observatory data should be confirmed in controlled trials to prove causality.

Within our *HOMe aFers* study, electrocardiographic, echocardiographic, and exploratory biomarker studies did not demonstrate clinical consequences of an FCM-induced temporary rise in iFGF23 with its subsequent urine phosphorus losses and low plasma 1.25 dihydroxyvitamin D. However, the limited number of participants may have affected our study’s power to detect bone and cardiac sequelae. In a larger trial comparing FCM and FDI at those dosages that are licensed in the USA, hypophosphatemia after FCM was accompanied by modest—albeit significant—changes in markers of bone resorption [9].

There are several limitations of this trial. First, the number of participants is limited, as we decided to stop the study after our pre-specified interim analysis yielded a significant difference in the primary endpoint between FCM and FDI. Although this decision followed our study protocol and a post hoc power analysis yielded a power of 92% for the primary outcome, completing a clinical trial after interim analyses always bears a risk to overestimate a treatment difference, which might have been mitigated if we had included more study participants. Moreover, as discussed before, the reduced number of study participants may have affected the power to discern cardiac and bone sequelae of FCM-induced hypophosphatemia. Next, intravenous iron was infused only once, so we cannot detect potential consequences from repeated infusions. Finally, the study period was limited to five measurements within 5 weeks, so that we cannot discern the exact time point at which plasma phosphorus, iFGF23, PTH, and 1.25 dihydroxyvitamin D returned to baseline values in each individual patient. Specifically, additional study visits between day 8 and week 5 would have yielded more granular information.

## Conclusion

In *HOMe aFers*, we found in women with uterine bleeding and iron deficiency anemia an incidence of hypophosphatemia after high-dose iron repletion with FCM that is similar to those reported in three earlier clinical trials [6, 8, 9]. Moreover, our study confirms that the high incidence of hypophosphatemia after FCM that occurs when applying dosages licensed in the USA [9] is virtually identical to those that will occur when applying dosages of FCM licensed in Europe.

Finally, we found that episodes of hypophosphatemia occur substantially less often after FDI than after FCM when given at equivalent dosages.

## Supplementary information

**Additional file 1: Table S1.** Exclusion criteria.

**Additional file 2: Figure S1A.** Screening, randomization and follow up.

**Additional file 3: Table S2.** Study’s curriculum.

**Additional file 4: Table S3.** Correlations between parameters of iron metabolism and bone and mineral metabolism.

**Additional file 5: Table S4.** Incidence of hypophosphatemia at different study timepoints in both treatment arms.

## Data Availability

The datasets generated and/or analyzed during the current study are not publicly available due to organizational reasons, but they are available from the corresponding author on reasonable request.
