# Supplementary material for: Hypophosphatemia after high-dose iron repletion with ferric carboxymaltose and ferric derisomaltose—the randomized controlled HOMe aFers study
Source: BMC Med. 2020 Jul 13;18:178. doi: 10.1186/s12916-020-01643-5 (PMC7359262; doi:10.1186/s12916-020-01643-5)
Supplement: Supplementary file 1 — Additional file 1: Table S1. Exclusion criteria. [file 12916_2020_1643_MOESM1_ESM.docx]

**Additional file 1: Table S1:** Exclusion criteria

- advanced chronic kidney disease, defined as estimated glomerular filtration rate (eGFR) according to the creatinine-based CKD-EPI equation ≤ 15 ml / min / 1.73 m² or renal replacement therapy
- hypophosphatemia, defined as plasma phosphate < 2.5 mg/dl at screening
- pregnancy or ongoing lactation
- known hypersensitivity to any intravenous iron preparations
- hemochromatosis
- untreated hyperparathyroidism
- active malignancy
- previous treatment with intravenous iron substitution within the previous 30 days or treatment with erythropoiesis stimulation agents (ESA)
- red blood cell transfusion within the previous 60 days
- radiotherapy or chemotherapy within the previous 60 days
- surgical procedures requiring general anesthesia within the previous ten days
- more than 1.5 times higher levels of alanine transaminase (ALT) or aspartate transaminase (AST) than normal range
- acute febrile inflammatory infection within the previous seven days
- systemic inflammatory diseases or diseases that need an anti-inflammatory treatment
- known bronchial asthma or known atopic dermatitis
- presence of relative contraindications (any allergy, any immunologic or inflammatory disease, history of atopic allergies), for which a treatment with the medical investigational products is not deemed indicated by the investigator
- women of childbearing potential without an effective method of contraception
- known active alcohol or drug abuse
- individuals with a history of a psychological illness or seizures
- individuals with known non-compliance
- administration of any investigational drug within the previous 30 days
