# Supplementary figures and images for: Hypophosphatemia after high-dose iron repletion with ferric carboxymaltose and ferric derisomaltose—the randomized controlled HOMe aFers study
Source: BMC Med. 2020 Jul 13;18:178. doi: 10.1186/s12916-020-01643-5 (PMC7359262; doi:10.1186/s12916-020-01643-5)

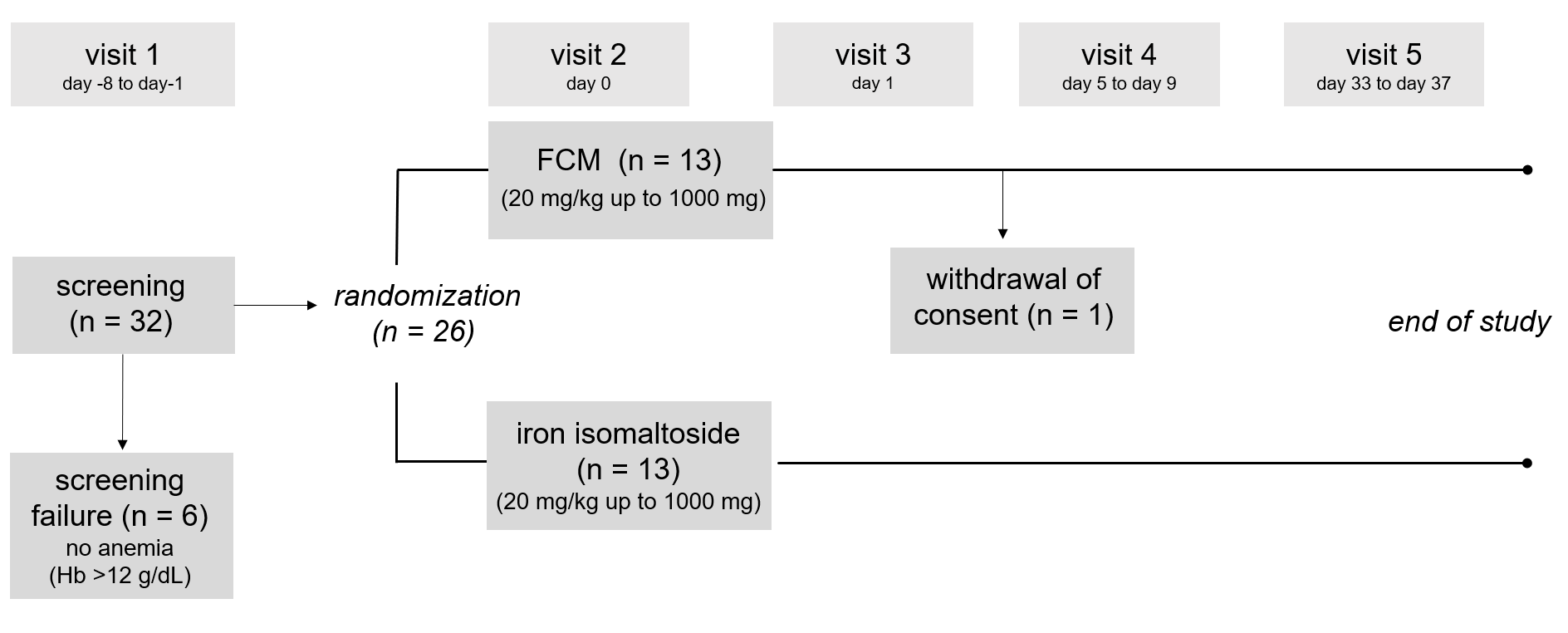


**Additional file 2: Figure S1A:** Screening, randomization and follow up

Supplement: Supplementary file 2 — Additional file 2: Figure S1A. Screening, randomization and follow up. [file 12916_2020_1643_MOESM2_ESM.docx]
