# Supplementary material for: Hypophosphatemia after high-dose iron repletion with ferric carboxymaltose and ferric derisomaltose—the randomized controlled HOMe aFers study
Source: BMC Med. 2020 Jul 13;18:178. doi: 10.1186/s12916-020-01643-5 (PMC7359262; doi:10.1186/s12916-020-01643-5)
Supplement: Supplementary file 3 — Additional file 3: Table S2. Study’s curriculum. [file 12916_2020_1643_MOESM3_ESM.docx]

| **Additional file 3: Table S2:** Study’s curriculum | | |
| --- | --- | --- |
| Visit number | time point | Data collection |
| 1 | day -8 to day -1 | documentation of medical history  physical examination  laboratory testing of blood and urine samples  test for pregnancy  12 lead electrocardiography, Holter ECG  ambulatory electrocardiography |
| 2 | day 0, before iron infusion (baseline) | physical examination  laboratory testing of blood and urine samples  12 lead electrocardiography  echocardiography  quality of life questionnaire (SF-36, Sheehan disability scale, MFI) |
| 2 | day 0 | ***iron infusion*** according to the randomization arm |
| 3 | day 1 | physical examination  laboratory testing of blood samples |
| 4 | day 5 to day 9 | physical examination  laboratory testing of blood and urine samples  12 lead electrocardiography, Holter ECG  ambulatory electrocardiography  echocardiography |
| 5 | day 33 to day 37 | physical examination  laboratory testing of blood and urine samples  12 lead electrocardiography  quality of life questionnaire (SF-36, Sheehan disability scale, MFI) |
| ***end of follow up*** | | |
| interim  visit | day 0 to day 37 | physical examination  laboratory testing of blood sample |
