# Supplementary material for: Hypophosphatemia after high-dose iron repletion with ferric carboxymaltose and ferric derisomaltose—the randomized controlled HOMe aFers study
Source: BMC Med. 2020 Jul 13;18:178. doi: 10.1186/s12916-020-01643-5 (PMC7359262; doi:10.1186/s12916-020-01643-5)
Supplement: Supplementary file 4 — Additional file 4: Table S3. Correlations between parameters of iron metabolism and bone and mineral metabolism. [file 12916_2020_1643_MOESM4_ESM.docx]

**Additional file 4: Table S3:** Correlations between parameters of iron metabolism and bone and mineral metabolism

|  |  | **1.25- dihydroxy- vitamin D** | **plasma**  **calcium** | **plasma**  **phosphorus** | **TSAT** | **ferritin** | **25-hydroxy- vitamin D3** | **parathormone** | **cFGF23** | **iFGF23** |
| --- | --- | --- | --- | --- | --- | --- | --- | --- | --- | --- |
| **1.25-dihydroxyvitamin D** | **r** |  | 0.068 | -0.522 | -0.130 | -0.266 | -0.007 | 0.335 | 0.289 | -0.036 |
|  | **p** |  | 0.747 | **0.006** | 0.526 | 0.190 | 0.974 | 0.095 | 0.162 | 0.863 |
| **plasma calcium** | **r** | 0.068 |  | -0.209 | -0.123 | 0.123 | 0.200 | -0.167 | -0.308 | 0.008 |
|  | **p** | 0.747 |  | 0.317 | 0.558 | 0.557 | 0.338 | 0.424 | 0.143 | 0.971 |
| **plasma phosphorus** | **r** | -0.522 | -0.209 |  | 0.292 | 0.173 | -0.041 | -0.191 | -0.285 | 0.066 |
|  | **p** | **0.006** | 0.317 |  | 0.147 | 0.399 | 0.843 | 0.351 | 0.167 | 0.754 |
| **TSAT** | **r** | -0.130 | -0.123 | 0.292 |  | 0.717 | 0.257 | -0.148 | -0.403 | -0.035 |
|  | **p** | 0.526 | 0.558 | 0.147 |  | **< 0.001** | 0.205 | 0.470 | **0.046** | 0.869 |
| **ferritin** | **r** | -0.266 | 0.123 | 0.173 | 0.717 |  | 0.451 | -0.238 | -0.305 | 0.025 |
|  | **p** | 0.190 | 0.557 | 0.399 | **< 0.001** |  | **0.021** | 0.242 | 0.138 | 0.906 |
| **25-hydroxyvitamin D3** | **r** | -0.007 | 0.200 | -0.041 | 0.257 | 0.451 |  | -0.310 | -0.060 | 0.383 |
|  | **p** | 0.974 | 0.338 | 0.843 | 0.205 | **0.021** |  | 0.124 | 0.777 | 0.059 |
| **parathormone** | **r** | 0.335 | -0.167 | -0.191 | -0.148 | -0.238 | -0.310 |  | 0.257 | -0.037 |
|  | **p** | 0.095 | 0.424 | 0.351 | 0.470 | 0.242 | 0.124 |  | 0.216 | 0.862 |
| **cFGF23** | **r** | 0.289 | -0.308 | -0.285 | -0.403 | -0.305 | -0.060 | 0.257 |  | 0.173 |
|  | **p** | 0.162 | 0.143 | 0.167 | **0.046** | 0.138 | 0.777 | 0.216 |  | 0.410 |
| **iFGF23** | **r** | -0.036 | 0.008 | 0.066 | -0.035 | 0.025 | 0.383 | -0.037 | 0.173 |  |
|  | **p** | 0.863 | 0.971 | 0.754 | 0.869 | 0.906 | 0.059 | 0.862 | 0.410 |  |

Indicated are univariable Pearson correlation coefficients. P-values in bold letters are significant. cFGF23 = c-terminal FGF23, iFGF23 = intact FGF23, TSAT = transferrin saturation
