# Supplementary material for: Hypophosphatemia after high-dose iron repletion with ferric carboxymaltose and ferric derisomaltose—the randomized controlled HOMe aFers study
Source: BMC Med. 2020 Jul 13;18:178. doi: 10.1186/s12916-020-01643-5 (PMC7359262; doi:10.1186/s12916-020-01643-5)
Supplement: Supplementary file 5 — Additional file 5: Table S4. Incidence of hypophosphatemia at different study timepoints in both treatment arms. [file 12916_2020_1643_MOESM5_ESM.docx]

| **Additional file 5: Table S4: Incidence of hypophosphatemia at different study timepoints in both treatment arms** | | | | |
| --- | --- | --- | --- | --- |
| Treatment arm | Visit 2 (Baseline) | Visit 3 (day 1) | Visit 4 (day 5 – 9) | Visit 5 (day 33 – 37) |
| FCM | 0 / 13 | 0 / 13 | 9 / 12 (75 %) | 3 / 12 (25 %) |
| FDI | 0 / 13 | 0 / 13 | 1 / 13 (8 %) | 1 / 13 (8 %) |

FCM = ferric carboxymaltose; FDI = ferric derisomaltose. One participant – randomized to the FCM treatment arm – withdrew her informed consent after visit 3, leaving 25 participants for analysis.
